# The Impact of Physical Therapy on Postural and Myotonometric Disorders in Patients with Pectus Excavatum: Study Protocol

**DOI:** 10.3390/life15101624

**Published:** 2025-10-17

**Authors:** Marius-Zoltan Rezumeș, Liliana Cațan, Elena Amăricăi, Ada-Maria Codreanu, Andreea-Ancuța Vătăman, Vlad-Laurentiu David

**Affiliations:** 1 Doctoral School, “Victor Babeș” University of Medicine and Pharmacy, 300041 Timișoara, Romania; marius.rezumes@umft.ro (M.-Z.R.); codreanu.ada@uvvg.ro (A.-M.C.); andreea.vataman@umft.ro (A.-A.V.); 2 Research Center for Assessment of Human Motion, Functionality and Disability, “Victor Babeș” University of Medicine and Pharmacy, 300041 Timișoara, Romania; amaricai.elena@umft.ro; 3 Department of Rehabilitation, Physical Medicine and Rheumatology, Faculty of Medicine, “Victor Babeș” University of Medicine and Pharmacy, 300041 Timișoara, Romania; 4 Department of Medicine, Faculty of Medicine, “Vasile Goldiș” Western University, 310038 Arad, Romania; 5 Department of Pediatric Surgery, “Victor Babeș” University of Medicine and Pharmacy, 300041 Timișoara, Romania; david.vlad@umft.ro

**Keywords:** data analysis, elasticity, evaluation, exercise therapy, muscle tone, pectus excavatum, posture, respiratory function tests, cardiovascular function tests

## Abstract

Pectus excavatum (PE) is the most common deformity of the chest wall seen in children and adolescents. Besides its visible depression of the chest, this condition often causes functional impairments affecting the cardiovascular and respiratory systems, as well as postural issues. Additionally, the aesthetic aspect of the deformity can greatly impact the psychosocial well-being of those affected. This study aims to evaluate the effect of a tailored physiotherapy program on children and adolescents with PE, focusing on musculoskeletal, cardiopulmonary, postural, and balance measures. A total of 35 participants diagnosed with PE will be assessed using four complementary methods: myotonometry with MyotonPRO for the trapezius muscle involving all three fascicles and the pectoralis major muscle on both sides, cardiopulmonary exercise testing on a treadmill (including cardiopulmonary function), postural assessment with GaitOn, and static balance-stabilometry with PoData 2.0. These assessments will be performed before and three months after completing an individualized physiotherapy program, which participants will perform daily after proper instruction from a physical therapist. After three months, the initial and final results will be compared to determine how physical therapy influences treatment outcomes in patients with PE.

## 1. Introduction

Pectus excavatum (PE) is the most common deformity of the anterior chest wall in children, with an average incidence of 1 in 1000 newborns [1]. PE occurs five times more frequently in males [1]. It involves a concavity of the sternum and the costal cartilages caused by unbalanced growth of the costochondral regions of the anterior chest wall [2]. The inward bending of the chest wall has two main effects. First, it reduces chest volume, leading to decreased vital capacity (VC), forced vital capacity (FVC), forced expiratory flow (FEF25-75%), and forced expiratory volume per second (FEV1). Second, it compresses the heart, primarily at the right ventricle and atria levels. Heart compression results in incomplete filling of its chambers, which lowers the blood volume ejected with each heartbeat [3]. However, most patients maintain sufficient cardiac output at rest or during minimal effort and only experience insufficiency during intense activity. This limitation is likely more related to effort capacity restrictions than to restrictive lung dysfunction. Depending on the severity of the deformity, PE symptoms can range from minor to significant cardiopulmonary impairment [4]. In addition to ventricular systolic dysfunction, various types of arrhythmias may also be present [4].

PE is often linked with multiple axial and thoracic deviations, such as an asymmetrically developed anterior thorax, drooping shoulders, thoracic kyphosis, and sometimes even thoracic scoliosis, which is a secondary form of scoliosis [5]. These features are significant because the deviations mentioned influence the entire biomechanics of the rib cage and, indirectly, the entire body by altering the geometry and kinematics of the rib cage [6]. In turn, the rib cage plays a vital role in spine biomechanics, serving as a sturdy framework for muscle attachment and development that supports the stability and mobility of the entire body [6].

PE patients can be treated through various methods, including surgery or complementary non-surgical approaches, depending on their age and the severity of their condition [7]. For much of the twentieth and twenty-first centuries, surgery was the main treatment option. However, recent decades have seen the development of new non-surgical techniques to address this condition [8]. PE is believed to have a multifactorial origin, potentially involving genetic predisposition, connections to connective tissue disorders (such as Marfan syndrome), and debated environmental factors [7].

Complementary methods of conservative treatment include negative pressure therapy, which involves applying a suction cup to the front part of the chest, generating negative pressure on the concave area. The procedure is performed daily for about 60–90 min over at least 18 months. Vacuum bell therapy offers a non-invasive option, especially effective in younger patients, although results vary and depend on compliance; long-term data are still limited [9,10].

Physical exercise is another method to address PE. For these patients, the exercise program should also incorporate specific exercises for respiratory rehabilitation to enhance exercise tolerance. Pulmonary re-education helps increase awareness of how to properly manage breathing when lung function begins to decline, and how to decompress the sternum from vital organs such as the heart and lungs [11].

It should be noted that PE is associated with a series of axial deviations, most commonly kyphosis and thoracic scoliosis, and physical exercise helps to correct and balance the muscles involved. Therefore, the patient will improve overall posture by working on the stability and mobility of the thoracic cage, rebalancing its geometry, with the muscles involved physiologically supporting the spine [12].

There are no studies on changes in the length and elasticity of the rib cage muscles, which might be involved in the development of pectus excavatum in children and adolescents. This lack of research is one reason why we chose to focus on this topic in our paper.

Most likely, a complex mechanism causes pectus excavatum, involving not only genetic factors but also changes in growth cartilages and faulty biomechanics of the rib cage, which is a hypothesis we propose to investigate [13].

The study aims to evaluate children and adolescents with PE using four methods: myotonometry (with MyotonPRO) for the Trapezius muscle, assessing all three fascicles; the Pectoralis Major muscle on both the left and right sides; cardiopulmonary exercise testing on a walking/running treadmill (with cardio-pulmonary exercise function); postural assessment (with GaitOn); and static balance-stabilometry (with PoData 2.0), conducted before and 3 months after an individualized physiotherapy program.

## 2. Materials and Methods

This protocol study aims to evaluate a group of patients with PE treated with physical therapy to highlight the importance of physical exercise in managing this condition. Respiratory rehabilitation helps restore muscle elasticity and corrects the overall body posture. This study is conducted at the Emergency Clinical Hospital for Children in Timișoara, within the Medical Rehabilitation Department, from March 2025 to July 2026.

This article outlines the study protocol for assessing the efficacy and safety of a structured physiotherapy program in children with pectus excavatum; we also note that this is a prospective study. The main objective is to evaluate the effect of the physiotherapy program on respiratory function and body posture after three months of treatment, while secondary objectives include safety monitoring, assessment of postural improvements, and analysis of quality-of-life outcomes. By publishing this protocol, we aim to promote transparency in research methodology and provide a clear framework for the upcoming clinical study, the results of which will be shared in a later article.

### 2.1. Sample Size Calculation

The effect size was 0.5, the type I error was α = 0.05, and power was 0.8. A total sample size of at least 35 patients was required. The sample size was calculated using G*Power 3.1.9.7 (Universitat Kiel, Kiel, Germany) with the Wilcoxon signed-rank test (matched pairs)

The within-group analyses will evaluate the effects over time. The evaluation of effect size was based on Cohen’s conventions for large effects [14]. The total sample size of our study (at least 35 patients) is in accordance with that calculated by Zielinski for patients who followed kinesiotherapy with the same statistical power and effect size (36 patients) [15].

### 2.2. Recruitment and Informed Consent

The study involves 35 patients with PE who wish to participate in a three-month physiotherapy program. Before the study begins, parents or guardians will be informed both verbally and in writing about all the details and milestones of the study.

The study is conducted in accordance with the Declaration of Helsinki and has been approved by the Ethics Committee of the “Victor Babeș” University of Medicine and Pharmacy in Timișoara, NO. 12/10.02.2025.

#### 2.2.1. Inclusion Criteria

The requirements for participation in the study are as follows: the subject diagnosed with PE must be a child or adolescent, not older than 18 years, and must exhibit a clinically visible appearance with a mild anterior chest concavity, confirmed by a CT scan using the Haller index, which should be less than 3.25. This index represents the ratio between the transverse diameter of the chest and its anteroposterior diameter at the point where the deformity is most pronounced. Lastly, participants must be available for the necessary evaluations at both the start and end of the study. [3]. Severity is usually quantified by the Haller Index and the Correction Index, both widely accepted for guiding treatment [3]. The Haller Index was developed in the 1980s and is based on CT images of the chest, calculated by dividing the transverse diameter by the sagittal diameter of the rib cage [16]. The Correction Index is a more recent and more precise tool for assessing the severity of PE, calculated by dividing the minimum distance between the posterior sternum and anterior spine by the maximum distance between the anterior spine and the most anterior portion of the chest [17].

#### 2.2.2. Exclusion Criteria

Subjects will be excluded from the study if they have had less than 3 months since any surgery, are recalcitrant, unable to concentrate, refuse cooperation, or are unwilling to follow the daily exercise plan. Children with mild scoliosis associated with the condition are eligible; they are evaluated during the initial consultation (clinical, paraclinical, and functional). Regarding associated pathology, the study participant must be free of neurological problems, epilepsy, moderate or severe scoliosis, or genetic syndromes.

#### 2.2.3. Discontinuing Criteria

Any subject who does not follow, interrupt, or deviate from the daily exercise program will be excluded from the study. Patients are also excluded if, during the study period, they suffer any injury that may affect their ability to adhere to the physiotherapy program.

### 2.3. Exercise Program

Following the parameters obtained during the initial assessment, the physiotherapist will develop a personalized exercise plan tailored to the patient’s needs.

At this stage, the physiotherapy program is designed and learned under the guidance of the physical therapist (2 weeks, 5 sessions per week, 60 min per session) and continued at home daily for three months. During the 10 physiotherapy sessions with the specialist, the patient carefully and correctly learns the exercises to perform at home in the next phase.

The objectives of the exercise program are to correct upper trunk posture, straighten the spinal column, increase mobility, re-educate muscles (by strengthening paravertebral muscles and relaxing muscles in the anterior thoracic area), and prevent or correct secondary axial deviations. Additionally, it aims to improve respiratory function and properly manage breathing during exercise.

The program includes exercises for spinal and rib cage mobility, exercises for toning the extensor trunk muscles (focusing on strengthening the back muscles and relaxing or stretching the front trunk muscles), respiratory re-education exercises, and exercises for stability re-education. To understand the program, Table 1 provides a detailed description of the exercises specifically designed by the authors in a potential work plan.

Proposed exercise plan for patients with PE and secondary axial deviations:

**Table 1:** Proposed exercise plan for patients with PE.

	Medical Rehabilitation Center	Home Exercise
Exercise Description	Number of Repetitions Week 1–2	Number of Repetitions Week 3–12
***Exercise 1:*** Patient sitting on a Bobath ball, feet on the ground, knee and hip joint, upper limbs placed behind the cervical spine with palms in contact with the cervical spine and arms close to the ear. The patient brings the arms back with an inhale, holds the position for 3–5 s and returns to the starting position with an exhale. (Figure 1a,b)	10 repetitions × 2 sets	10 repetitions × 3 sets
***Exercise 2:*** The patient on the Bobath ball, seated as in the previous exercise, positions the hands under the mandible parallel to the ground, and brings the arms backwards with double extension while inhaling, and then forward while exhaling.	10 repetitions × 2 sets	10 repetitions × 3 sets
***Exercise 3:*** The patient on the Bobath ball, seated as in exercise 1, positions the hands on the shoulders and to perform the exercise brings the arms backwards, forming an imaginary circle, when the arms are parallel to the ground, maintains the position for 3–5 s and returns to the initial position continuing the imaginary circle. (Figure 2a,b)	10 repetitions × 2 sets	10 repetitions × 3 sets
***Exercise 4:*** The patient, sitting with the buttocks on the heels, stretches forward with the hands on the Bobath ball and does trunk extension to stretch the anterior thoracic muscles.	10 repetitions × 2 sets	10 repetitions × 3 sets
***Exercise 5:*** The patient in standing position, upper limbs pointing forward (shoulder flexion 90°), with a 0.5 kg dumbbell in each hand, the exercise requires the sideways movement of one arm at a time, inhaling and back exhaling (Figure 3a,b) an update of the exercise would be to simultaneously extend the arms.	10 repetitions × 2 sets	10 repetitions × 3 sets
***Exercise 6:*** Patient in orthostasis with a cane above the head, positioned horizontally between the two hands, lower the cane to the back pressing on the shoulder blades while inhaling, and back while exhaling.	10 repetitions × 2 sets	10 repetitions × 3 sets
***Exercise 7:*** Patient in orthostasis with a cane positioned horizontally between the two hands, raises the cane overhead while inhaling, and back while exhaling. (Figure 4a,b)	10 repetitions × 2 sets	10 repetitions × 3 sets
***Exercise 8:*** Patient in orthostasis, arms extended parallel to the floor, the exercise involves extending the trunk while lifting the arms and legs off the floor.	10 repetitions × 2 sets	10 repetitions × 3 sets
***Exercise 9:*** Patient in prone position, arms stretched, holding a cane, the exercise involves pulling the cane to the chest with slight extension of the spine + inhaling, return to the initial position on exhaling (Figure 5a,b)	10 repetitions × 2 sets	10 repetitions × 3 sets
***Exercise 10:*** Aerobic training: stationary cycling in order to increase exercise tolerance, increase energy intake.	15 min	20 min

### 2.4. Measurements

In the first stage, we conduct investigations to establish the patient’s sample, and the physiotherapist designs a personalized physiotherapy program. This is followed by the second stage, where the physiotherapy program is carried out and learned under the guidance of the specialist. The final stage involves re-evaluating the patient after completing the physiotherapy program and drawing conclusions from the study.

Each parameter will be reviewed by the investigators, documented in an Excel file, and then their correlation will be analyzed using a statistical program.

In the initial phase after establishing the two samples, evaluation of each subject begins as follows: myotonometric assessment of the involved muscles (Trapezius muscle with all three fascicles, Pectoralis Major muscle), on both the left and right sides, along with cardiopulmonary exercise testing (CPET), postural analysis (GaitOn), and static balance measurement (stabilometry-PoData), using non-invasive tests.

#### 2.4.1. Myotonometry

The myotonometer offers an objective method to measure the physiological properties of muscle tone. The MyotonPRO is a compact, portable, non-invasive myotonometer that provides an objective measurement of the mechanical properties of muscle [18].

The device simultaneously measures or calculates five parameters, divided into three sections: the first section assesses the muscle’s tension state by measuring the natural oscillation frequency [Hz], which reflects the muscle’s tension. The second section includes biomechanical properties such as dynamic stiffness [N/m] and logarithmic decrement, which characterize muscle elasticity. The third section covers viscoelastic properties of the muscle, with the device recording results on the mechanical stress relaxation time [ms] and the ratio of relaxation time to deformation time, indicating CREEP (Deborah number) [19].

The device is set for a specific target group and follows an internal protocol based on the muscles involved in the test. After introducing a new subject, the established protocol is loaded onto the device to begin the test.

**Figure 6:**
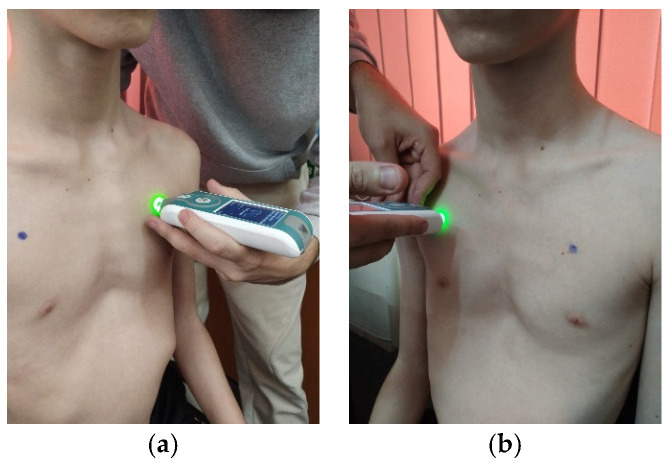
Myotonometric assessment of the left (**a**) and right (**b**) pectoralis major muscle.

**Figure 7:**
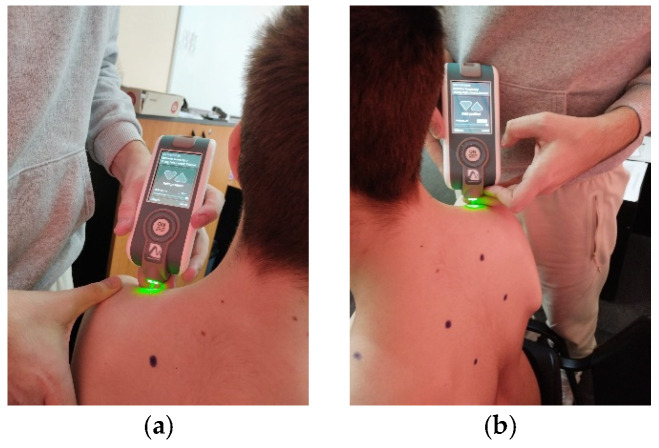
Myotonometric assessment of the left (**a**) and right (**b**) upper trapezius muscle.

**Figure 8:**
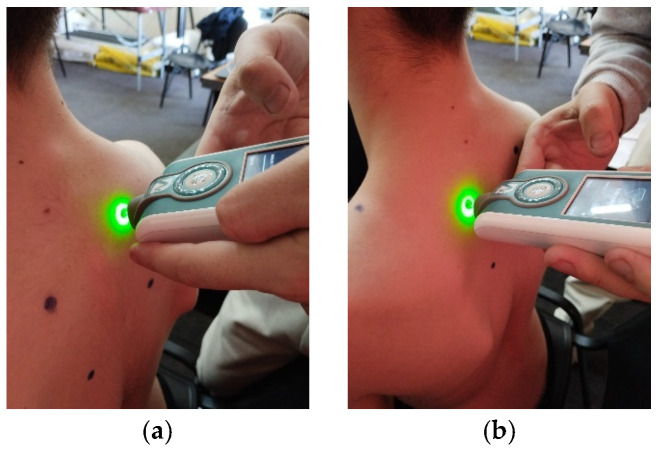
Myotonometric assessment of the left (**a**) and right (**b**) trapezius medius muscle.

**Figure 9:**
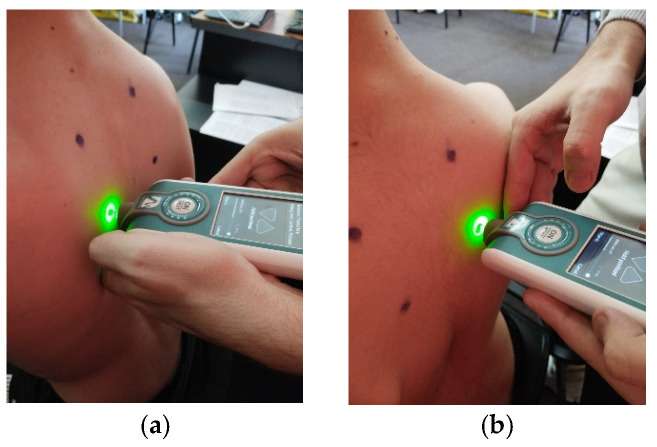
Myotonometric assessment of the left (**a**) and right (**b**) lower trapezius muscle.

For testing, the patient must be seated on a chair, and according to the established protocol, testing is performed bilaterally, always starting with the left side in the following order: pectoralis major (Figure 6a,b, measured 5 cm above the nipple), upper trapezius (Figure 7a,b, perpendicular to the muscle fibers at the midpoint between the lateral cervical line and the acromion), middle trapezius (Figure 8a,b, halfway between the spine and the medial border of the scapula at T3 level), and lower trapezius (Figure 9a,b, 3 cm medial and inferior to the inferior angle of the scapula, on the line between the scapula and the spine). To perform the test, touch the external end of the device to the central part of the muscle belly and wait for five direct pulses (each indicated by a green light). If the results are written in red, repeat the test on that muscle until the results turn white. After completing the test, upload the results to the laptop and then export them to a separate Excel document for statistical analysis.

#### 2.4.2. Cardiopulmonary Exercise Testing (CPET)

Assessment of cardiopulmonary condition will be performed with the BTL-CPET device, which uses glue-wired electrodes that are attached by a cable to a portable EKG device mounted around the patient’s abdomen [20].

Blood pressure should be measured at the beginning and at the end of the test. Alternatively, a blood pressure cuff may be attached to the patient’s arm. Additionally, a pulse oximeter will be worn on the patient’s finger to monitor tissue oxygen levels and heart rate.

A mask that attaches to the head with a rubber band is used for lung testing. It must be sealed so the person breathes only through the opening in the mask. A cylinder is inserted into the hole in the mask and connected by wires to an air filter. The filter provides data on the amount of oxygen inhaled and the amount of carbon dioxide exhaled.

**Figure 10:**
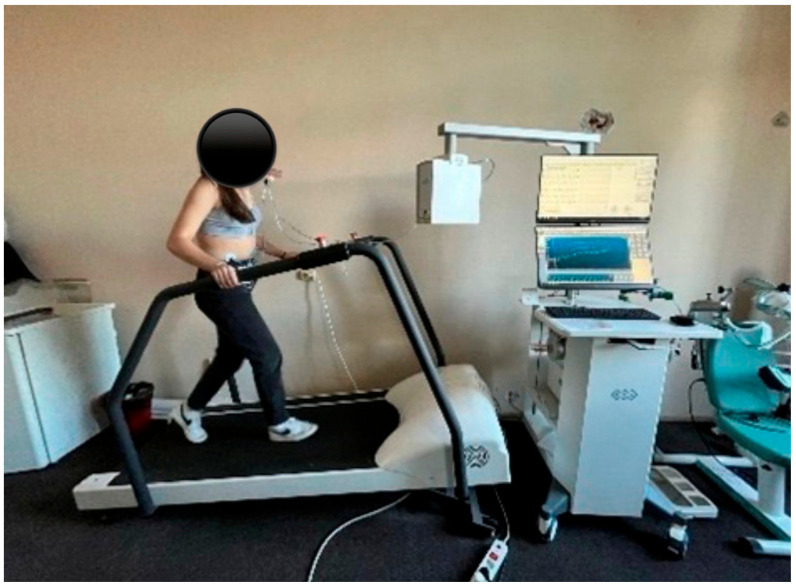
Cardiopulmonary exercise assessment.

Once the preparations are complete, the patient is asked to step onto the treadmill to start testing. This begins with a 3 min warm-up period during which the patient is asked to walk. After the warm-up, the actual testing begins. Each level has a specific incline and speed, which increase gradually during the test (Figure 10).

The study uses the modified Bruce Protocol, which starts at a speed of 1.7 mph and a 0% incline. The second and third stages maintain the same speed, but the slope increases by 5% in each stage. Each subsequent stage increases in speed by 1.12 to 1.28 km/h and in incline by 2%. The test lasts approximately 10 to 13 min, followed by a 3 min recovery period [21].

With cardiopulmonary exercise testing, we gather data on the body’s response to both maximal and submaximal exercise by recording cardiac parameters (electrocardiogram) and respiratory parameters. Depending on the respiratory gases, we calculate VE and VO_2max_ [22].

Endurance capacity indicators include maximal oxygen consumption (VO_2_) and ventilation thresholds (VT1 and VT2). Additionally, anaerobic capacity, movement economy, and mechanical efficiency can also be assessed through gas exchange measurements, while cardiopulmonary exercise testing helps define exercise intensity zones.

#### 2.4.3. Static Balance

**Figure 11:**
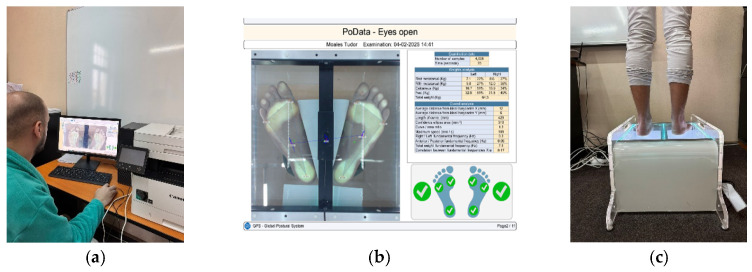
Static balance assessment (**a**–**c**) (personal collection, patient consent).

Stabilometric assessment will be conducted using the Chinesport device (PoDATA 2.0) [23]. The device measures body mass distribution at three points for each foot: the first and fifth metatarsal heads and the heel (Figure 11a–c). For the assessment, the patient is asked to remove their shoes. After the device is calibrated, the patient walks on the test surface, maintaining upright posture and performing various movements as directed by the coordinator. The evaluation includes the following tests, each lasting 20 s: eyes open, eyes closed, head rotated to the right or left, head tilted laterally to the right or left, and head in hyperextension. Each test is performed to observe how the center of gravity balances during different movements performed throughout the day, helping us understand how the PE affects stability.

#### 2.4.4. Evaluation of Posture (GaitOn)

Evaluation of patients’ posture and body alignment will be carried out using the GaitON Posture Analysis System, which assesses the patient’s posture from anterior, posterior, right, and left views. It identifies key postural deviations from multiple angles and calculates a ratio of the deviations [24].

**Figure 12:**
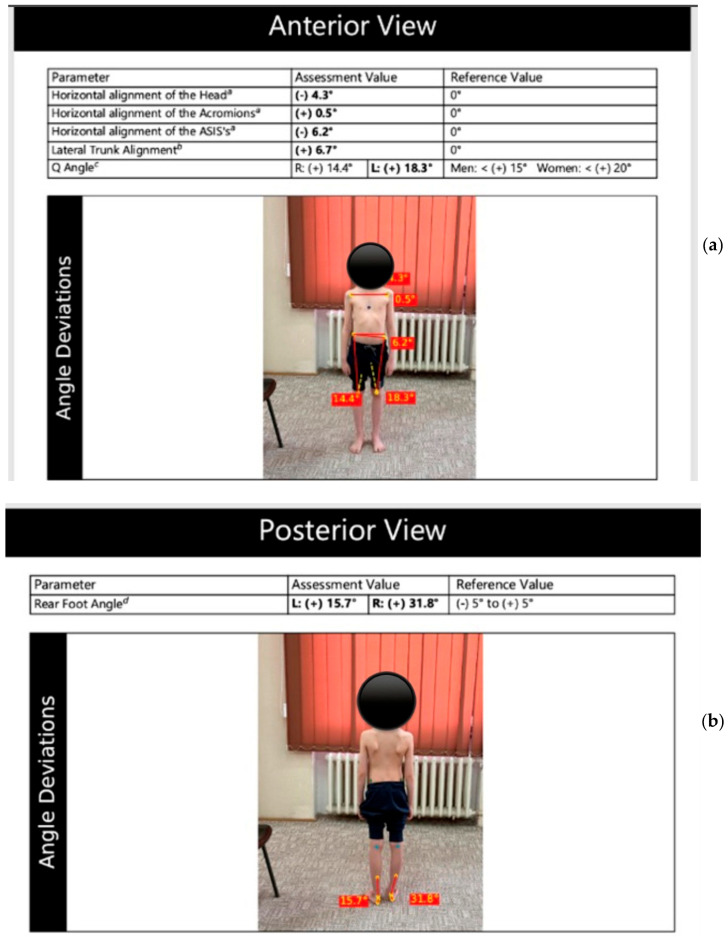
Evaluation of posture in anterior (**a**) and posterior view (**b**)—GaitOn (personal collection, patient consent).

**Figure 13:**
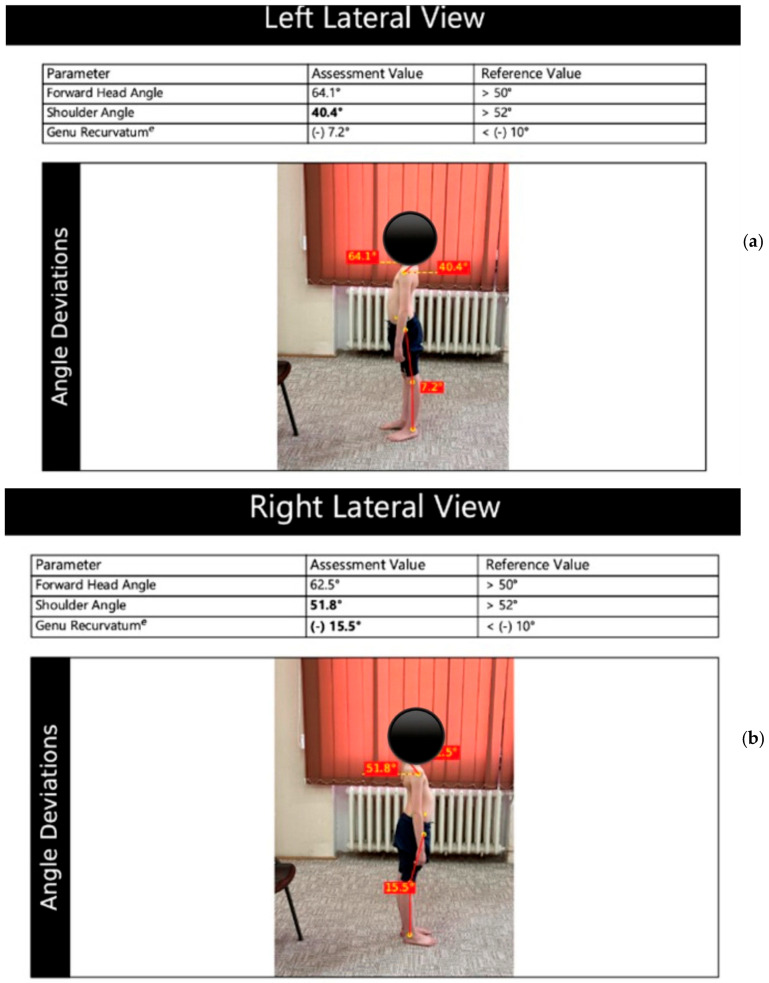
Evaluation of posture in left (**a**) and right lateral view (**b**)—GaitOn (personal collection, patient consent).

The patient is evaluated from four perspectives. Anterior view: Markers are placed at the earlobe, anterior superior iliac crest, midpoint of the patella, and on the tibial tuberosity, on both the right and left sides (Figure 12a). Posterior view: Markers are placed at the base of the calcaneus, where the Achilles tendon inserts, the center of the Achilles tendon, and mid-calf. (Figure 12b) Lateral left-right view: Markers are positioned at the C7 spinous process, midpoint of the humeral head, greater trochanter of the femur, lateral epicondyle of the femur, and lateral malleolus (Figure 13a,b).

To test after applying the markers, the patient is photographed from four angles (anterior, posterior, and lateral views on both the left and right sides). The photographs are entered into a system, and after analysis, a report is generated documenting and illustrating the associated axial deviations involved [24].

### 2.5. Statistical Analysis

Statistics will be performed using GraphPad Prism 5.0 for Windows. The descriptive statistics will be computed for all variables (mean and standard deviation). Before statistical applications, the normal distribution of values will be verified by the D’Agostino-Pearson normality test. The intragroup data (analyzed parameters at baseline and after the physical exercise program) will be compared with the paired *t*-test.

For the myotonometry, frequency, stiffness, decrement, stress relaxation time and creep of the trapezius and major pectoralis will be compared before and after the 3-month physical exercise program. VE and VO_2max_ will be analyzed at baseline and after the physical exercise program. Paired *t*-tests will be used to compare the intragroup data of the postural stability parameters (stabilometry data in the different testing situations). GaitOn postural analysis parameters will be measured before and after the 3-month physical exercise program.

## 3. Expected Results

Based on the hypothesis of our study, we expect changes in the parameters of the four assessments (myotonometry, stabilometry, postural analysis, and cardiopulmonary exercise testing) when comparing baseline data with the 3-month results. A difference in myotonometric parameters is anticipated between the concave (anterior thoracic) muscles and the Trapezius muscle, specifically in resting tone, stiffness, elasticity, and muscle relaxation time. Additionally, differences between the initial and 3-month evaluations are expected for the biomechanical and viscoelastic properties of the Trapezius and Pectoralis muscles.

We also expect differences between the baseline and the 3-month assessment in the GaitOn analysis parameters. Changes are anticipated in the lateral view examinations. The shoulder angle, with improved values, is targeted after the 3-month exercise program.

In terms of cardiopulmonary assessment, significantly positive differences are expected regarding the patient’s ability to manage breathing times (inhale and exhale) during exercise.

From a clinical perspective, we expect an overall improvement in the appearance and function of the chest, better posture, and potentially relief from some symptoms related to the condition.

Regarding stabilometry, the goal is to understand the differences and possible correlations of the resulting values after correcting posture and shifting the center of gravity. We anticipate improved postural stability in patients following the 3-month physical exercise program.

## 4. Discussion

The study will be randomized and will examine the characteristics of the trapezius and pectoralis major muscles, respiratory function, stability, and overall posture in children and adolescents with PE before and after a tailored physical therapy program lasting three months. The assessment of study participants will include four different testing methods: myotonometry, cardiopulmonary exercise testing (CPET), posture evaluation, and static balance analysis.

An excessively high position in the upper chest area may cause PE, and the pectoralis major muscle has a biomechanical role in lifting the sternum [25]. Consequently, the main hypothesis of this investigation will focus on assessing the tone of the pectoralis major and trapezius muscles, as well as examining any recorded discrepancies that might contribute to this condition. The study will explore changes in length, elasticity, muscle tone, and relaxation in muscles potentially involved in the development of the pathology through a non-invasive measurement method: myotonometry. No studies have been found in the literature that evaluate the properties of muscles involved in the development of PE. However, some evidence links thoracic kyphosis with this condition [6], prompting an analysis of the literature’s reported changes and a comparison with the findings of this study. Thoracic kyphosis appears as limited mobility of the thoracic spine caused by alterations in both non-contractile and contractile tissues [26].

Therefore, in a study conducted by Yeo et al., muscle tone was evaluated in the upper trapezius and pectoralis major muscles of patients with thoracic kyphosis using MyotonPro^®^ (Myoton AS, Tallinn, Estonia). Variations in the examined parameters were observed [27]. The measurement technique used in this study is similar to that in our research, with the MyotonPRO^®^ tip positioned vertically at the midpoint of the subjects’ upper trapezius and pectoralis major muscles.

The CPET evaluation of the study participants will assess the effect of sternal deformity on cardiopulmonary function and compare the results with those reported in existing literature.

Similarly to our study, Tariq Abu-Tair et al. evaluated these factors in 2018 through a study involving 99 children with pectus excavatum using CPET on a treadmill. Based on the measured Haller index, the results of CPET testing in children with mild and moderate pectus excavatum showed that cardiopulmonary function depends more on the individual’s level of exertion, and that cardiopulmonary capacity varies widely, from normal to moderate. Additionally, even among patients with a Haller index below 3.25, fewer than 15% exhibited moderate functional impairment of the cardiopulmonary system. The study’s findings indicated that the severity of sternal deformity correlates with its effect on cardiopulmonary function, with variations observed in heart rate and cardiac output. Specifically, there is an initial increase in heart rate at the anaerobic threshold, followed by a decline in stroke volume at both the anaerobic threshold and at maximum stroke volume, ultimately leading to a decrease in cardiac output due to the severity of the sternal deformity [28].

An abnormally low maximum anaerobic VO_2_ during exercise testing is a common finding reported by other studies that have documented cardiac dysfunction due to pectus excavatum. Using a treadmill for cardiopulmonary exercise testing, patients with pectus excavatum could not reach the vascular volumes of healthy control subjects at any exercise intensity. Furthermore, their low cardiac output limited their maximum exercise capacity [29,30].

Tiffany J. Zens et al. assessed 261 patients with pectus excavatum through cardiopulmonary exercise testing (CPET) using an ergometric bicycle, revealing that 33% of patients showed reduced aerobic capacity. The study found that the severity of PE correlates with dysfunction of both right and left ventricular systolic function and may also impact exercise tolerance [31].

Using an ergometric bicycle to perform CPET on 259 subjects with pectus excavatum and an average age of 15.8, [32] study asserts that ventilation disorders are not associated with exertional dyspnea or decreased aerobic fitness in patients with this deformity.

Investigating these aspects and comparing the results with those reported is motivated by the similarities and differences observed in CPET testing in children with pectus excavatum in the studies mentioned earlier, regardless of whether the tests were performed on an ergometric bicycle or a treadmill.

The GaitOn posture assessment is among the evaluation tools used in this study. Current practice notes that children and adolescents with PE also experience posture issues in their limbs and spine. Published studies support these observations, showing that these children tend to have a lower body weight [32] and poorer posture [33,34]. It is advisable to assess the spine for thoracic kyphosis, lumbar lordosis, pelvic tilt, and lateral tilt angles in the thoracic, lumbar, and pelvic regions, then refer patients to medical rehabilitation centers for correction [34]. The evaluation of posture in children and adolescents with PE is expected to show improvement in the identified parameters after three months of physical therapy.

Since no information on these aspects has been found in the literature, the evaluation of static balance (stabilometry-PoData) will be the fourth method used in this study to assess children with pectus excavatum.

Static balance in children with thoracic hyperkyphosis is affected by removing specific visual input and is linked to changes in the contraction and strength of certain trunk and lower extremity muscles [35].

Contrary to these observations, there are studies on young individuals with postural thoracic kyphosis that show no significant relationship between body balance and thoracic kyphosis [36]. These aspects will be assessed and analyzed in the current study. To help design an individualized exercise program and support one of the established conclusions, the static balance parameters of children and adolescents with PE at the end of the physical therapy program will be compared with the initial parameters, and the results will be analyzed.

A combined exercise program effectively corrects spinal deviations [37] and improves balance in adolescents with postural kyphosis [38].

Davi DE Podestá Haje et al. also highlight the importance of physical activity in children and adolescents with mild to moderate PE. A study involving participants who performed targeted exercises to strengthen the anterior chest muscles at least five times a week, under the supervision of a physical therapist and with controlled breathing, considered various factors, including patient age and compliance with the program. The results demonstrated the exercises’ effectiveness in correcting or partially correcting pectus excavatum, especially when therapy began early in cases of milder and more flexible deformities. The exercise regimen used by participants included simultaneous resistance-free abduction and extension of the upper limbs, trunk extension while lying down, push-ups, sit-ups, and blowing up a balloon for 10 min [39].

In a 2020 study, Nuray Alaca et al. recommended kinetotherapy, which includes cardiopulmonary exercises and chest muscle toning, for patients with pectus excavatum. The physical therapy program involved breathing exercises for each lung lobe, upper and middle/lower diaphragmatic lateral rib breathing exercises, and exercises for the musculoskeletal system that included postural awareness, trunk mobilization and manipulation, stretching, strengthening, and additional aerobic exercises [11].

The proposed exercise program, although similar to those described in previous studies and detailed in the Materials and Methods section, is unique because it is performed daily for three months and tailored individually based on parameters identified during the initial assessments.

The effect of the three-month exercise program on children and adolescents with PE will be studied in relation to deformity progression. The evaluated parameters will be correlated, and the findings compared with existing research to aid in developing an assessment and rehabilitation protocol for these patients.

## 5. Conclusions

This study aims to provide a variety of options for treating patients with PE after complex non-invasive evaluations. The results obtained will help us to detail how physiotherapy influences the treatment of patients with PE and how multidisciplinary cooperation can be recommended in the approach for this disease. Thus, we will consider proposing a protocol for the evaluation and treatment of children and adolescents with PE.

## Figures and Tables

**Figure 1:**
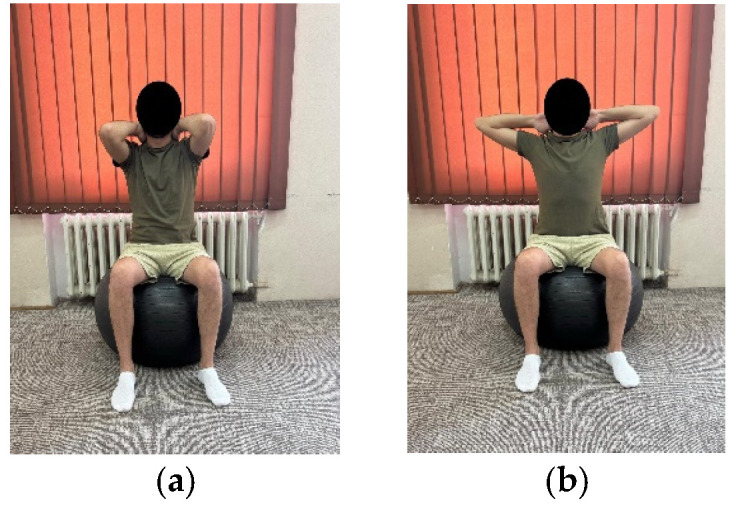
Exemplification of exercise 1: (**a**) initial position; (**b**) final position.

**Figure 2:**
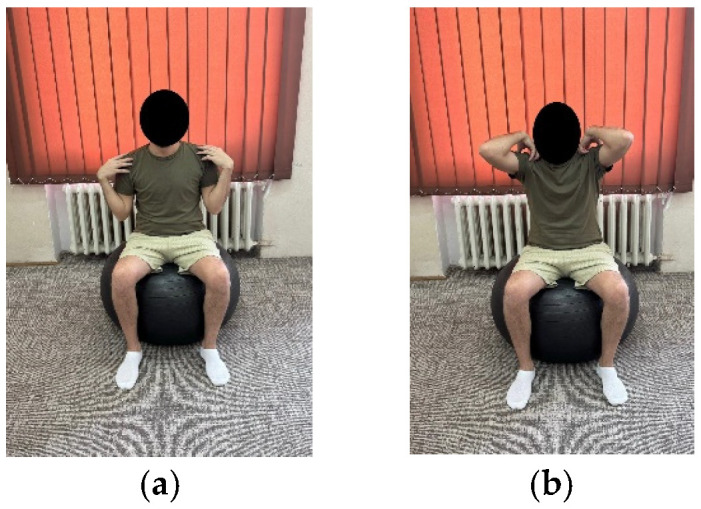
Exemplification of exercise 3: (**a**) initial position; (**b**) final position.

**Figure 3:**
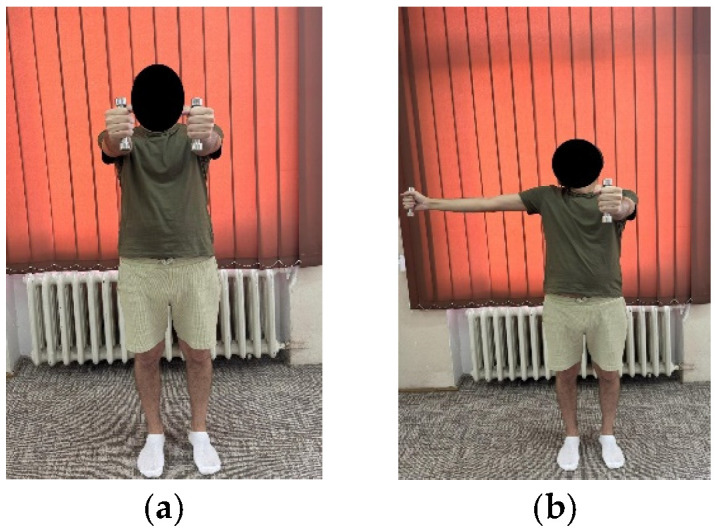
Exemplification of exercise 5: (**a**) initial position; (**b**) final position.

**Figure 4:**
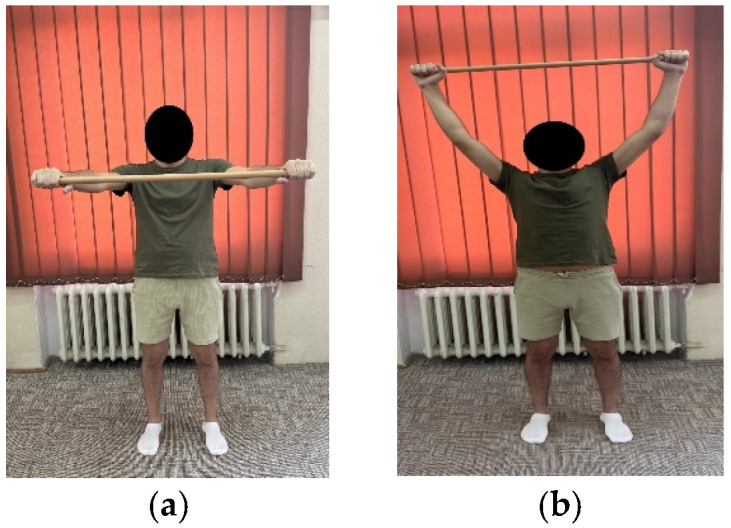
Exemplification of exercise 7: (**a**) initial position; (**b**) final position.

**Figure 5:**
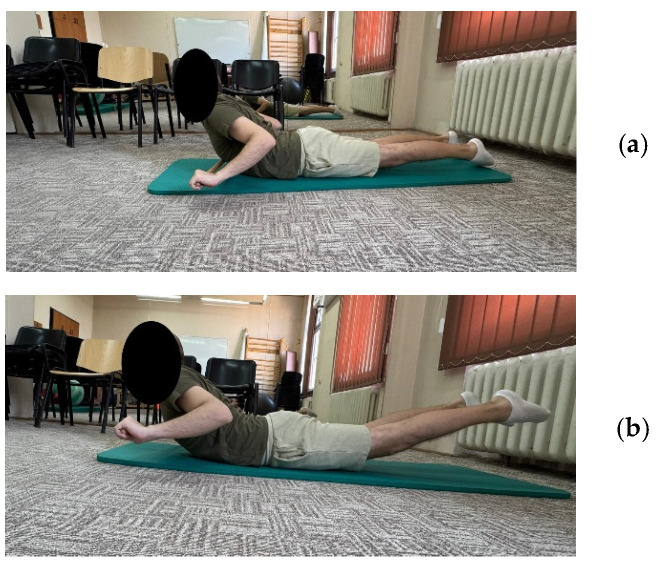
Exemplification of exercise 9: (**a**) initial position; (**b**) final position.

## Data Availability

The data presented in this study are available on request from the corresponding author (L.C.) due to privacy.

## Abbreviations

The following abbreviations are used in this manuscript:

PE	pectus excavatum
CPET	cardiopulmonary exercise testing
CT	computer tomography
EKG	electrocardiogram
VC	vital capacity
VE	ventilatory power
VO_2max_	maximal oxygen consumption
VT1	ventilatory threshold 1
VT2	ventilatory threshold 2
FEF	forced exhaling flow
FEV1	forced exhaling volume per second
FVC	forced vital capacity
